# The impact of antenatal care on under‐five mortality in Ethiopia: a difference‐in‐differences analysis

**DOI:** 10.1186/s12884-020-03531-5

**Published:** 2021-01-10

**Authors:** Samuel Oduse, Temesgen Zewotir, Delia North

**Affiliations:** School of Mathematics, Statistics and Computer Science, University of KwaZulu-Natal, 4001 Durban, South Africa

**Keywords:** Antenatal care, Difference‐in‐difference, Propensity score matching, Sustainable development goal, Under‐five mortality

## Abstract

**Background:**

Sub-Saharan Africa, as opposed to other regions, has the highest under-five mortality rates yet makes the least improvement in reducing under-five mortality. Despite the decline, Ethiopia is among the top ten countries contributing the most to global under-five mortalities. This article examines the impact of the number of antenatal care and the timing of first antenatal care on child health outcomes. We specifically investigated if the utilization of antenatal care services positively affects the reduction of under-five mortality.

**Methods:**

We employ a difference-in-differences design with propensity score matching to identify direct causal effects of antenatal care on under-five mortality based on the Ethiopian Demographic Health Survey data of 2011 and 2016. Our sample includes 22 295 women between the ages of 14–49 who had antenatal care visits at different times before delivery.

**Results:**

The study revealed 1 481 cases of reported under-five mortality. 99.0% of that under-five mortality cases are women who had less than eight antenatal care visits, while only 1% of that is by women who had eight or more antenatal care visits. Antenatal care visit decreases the likelihood of under-five mortality in Ethiopia by 45.2% (CI = 19.2–71.3%, P-value < 0.001) while the timing of first antenatal care within the first trimester decreases the likelihood of under-five mortality by 10% (CI = 5.7–15.6%, P-value < 0.001).

**Conclusions:**

To achieve a significant reduction in the under-five mortality rate, Intervention programs that encourages more antenatal care visits should be considered. This will improve child survival and help in attaining Sustainable Development Goal targets.

## Background

Child mortality is one of the critical indicators of health status in a country [1]. The international community has come up with several plans to improve child health outcomes. Governments have keyed into these plans to achieve necessary improvements in their communities’ health and general well-being.

By implementing comprehensive health development strategies, Ethiopia met the Millennium Development Goal 4, with the under-five mortality rate per 1000 live births decreasing from 205 to 1990 to 67 in 2015 [2, 3]. Despite this remarkable progress, the country’s under-five mortality rate still ranks among the highest in the world. An estimated one in every 15 children dies before reaching their fifth birthday in Ethiopia currently [4]; this presents a challenge to the government in achieving the Sustainable Development Goal (SDG). The SDG aims to reduce neonatal mortality to at least as low as 12 deaths per 1,000 live births and under-five mortality to at least as low as 25 deaths per 1,000 live births in all countries by 2030 [5].

Antenatal care (ANC) is a maternal health program provided by trained health workers to pregnant women. ANC’s primary objective is to identify and monitor pregnant women at high risk early [6]. The ANC offers risk recognition, prevention, and control of pregnancy-related diseases and health education for mothers and children [7].

Contact with health workers during ANC visits allows early dictation and treatment of health complications and pregnancy-related infections for pregnant women [8]. The ANC enables pregnant women to prepare for childbirth and learn and understand risk indicators in pregnancy. It also serves as an access point where pregnant women can get vitamin and mineral supplements essential for their well-being. When pregnant women contact health professionals during ANC appointments, they can receive relevant immunization and HIV tests. If a woman test positive, they will be trained and given medicine to avoid mother-to-child transmission.

The antenatal period also affords avenues for providing pregnant women with various interventions critical to their health. Evidence indicates that no nation has succeeded in bringing the maternal mortality ratio below 100 per 100 000 live births without ensuring that all women are attended by an adequately trained health professional during delivery and soon after birth [9].

The ANC guideline by WHO in 2016 suggests a minimum of eight ANC contacts, with the first contact expected to take place within the first trimester of gestation, this is to be followed by two visits in the second trimester and five contacts in the third trimester [10]. This newly expanded recommendation from the required four or more visits to at least eight contacts shows that ANC is not just about the number of visits but also about the timing of the first ANC contact and service quality.

In Ethiopia, ANC service utilization has improved, but many women still give birth at home without considering such services [11–13]. A recent study in Ethiopia shows the prevalence of ANC utilization to be 62.8% [14]. Another research also shows that only 6% of Ethiopian women make their first ANC visit within 16 weeks after gestation [15]. The Ethiopian government has introduced results-oriented measures to reduce maternal mortality and improve child health [16]. Such interventions include Community-based health extension workers and maternity waiting homes where women nearing their delivery date get temporarily accommodated who would have had difficulty getting to a health facility on time [17].

Several studies have shown ANC to have a positive effect on child mortality reduction [18–22]. Also, as a determinant of child mortality, ANC is strongly related to socioeconomic and environmental characteristics [23–25]. Therefore, we cannot overemphasize the importance of ANC visits for the well-being of the mother and the child after delivery. Past studies on ANC have often been limited to the association between ANC and child mortality. Therefore, this article contributes to the literature by estimating ANC’s contribution to Ethiopia’s health outcomes between 2011 and 2016. Specifically, we evaluated the impact ANC has on under-five mortality. We hope to determine if the number of ANC visits and the first ANC visit’s timing affects child health. We used a matching approach combined with a Difference-in-Difference research design to tackle confounding variables linked to child mortality and ANC.

## Methods

### Data and sample

This study is based on data from the 2011 and 2016 Ethiopian Demographic and Health Survey (EDHS). The EDHS are five-year periodic national representative household surveys that collect retrospective information on a wide variety of health, socioeconomic, and demographic factors for the population across all regions to improve maternal and child health in Ethiopia. The 2011 and 2016 EDHS used a stratified two-stage cluster sampling plan to select respondents for the study. Elaborate details of each survey protocols and their designs have been reported elsewhere [23, 24]. The EDHS’s information was obtained through personal interviews with women in the child-bearing age 15–49 years. The EDHS consist of three components: the household questionnaire, the woman’s questionnaire, and the man’s questionnaire. From the woman’s questionnaire, data for child mortality, along with related variables, were extracted. Based on mothers’ birth history information, the authors examined a combined sample of 11,654 children from the 2011 EDHS and 10,641 from 2016.

### Treatment and outcome

Our study’s health outcome is under-five child mortality, which refers to children’s death before reaching the age of five. The information is captured through the full birth’s history recalled by the interviewed women and recorded in the surveys. We define treatment as the utilization of antenatal care.

We have two sets of treatment; one is the number of ANC visits, and the second is the timing of the first ANC visit. For the first set, women who had only one ANC visit are considered as Treatment 1; they are compared to women who had no ANC visit (Control 1) throughout their entire pregnancy. Treatment 2 is women who had two ANC visits, and they were compared to women with no ANC visit or with only one visit (Control 2). Treatment 3 is women who had four ANC visits during their pregnancy, and they were compared to women who had less than four visits (Control 3). And Treatment 4 is women who had eight or more visits according to WHO recommendation, and they were compared with women who had less than eight visits for under-five mortality.

For the timing of the first ANC visit, women who had their first visit in the third trimester of gestation (Treatment 5) were compared to women who had no ANC visit (Control 5). Treatment 6 is women who had their first ANC visit in their second trimester; they were compared to women in the third trimester or had no visit (Control 6). Finally, women who had their first visit in the first trimester of gestation (Treatment 7) were compared to women who had their first ANC visit later than the first trimester. The rate of under-five mortality from 2011 to 2016 for the control groups will vary due to several possible unknown factors. The variation of this rate at the treatment groups will be due to the same factors plus the variation in the number of ANC visits or timing to the first ANC.

**Fig. 1:**
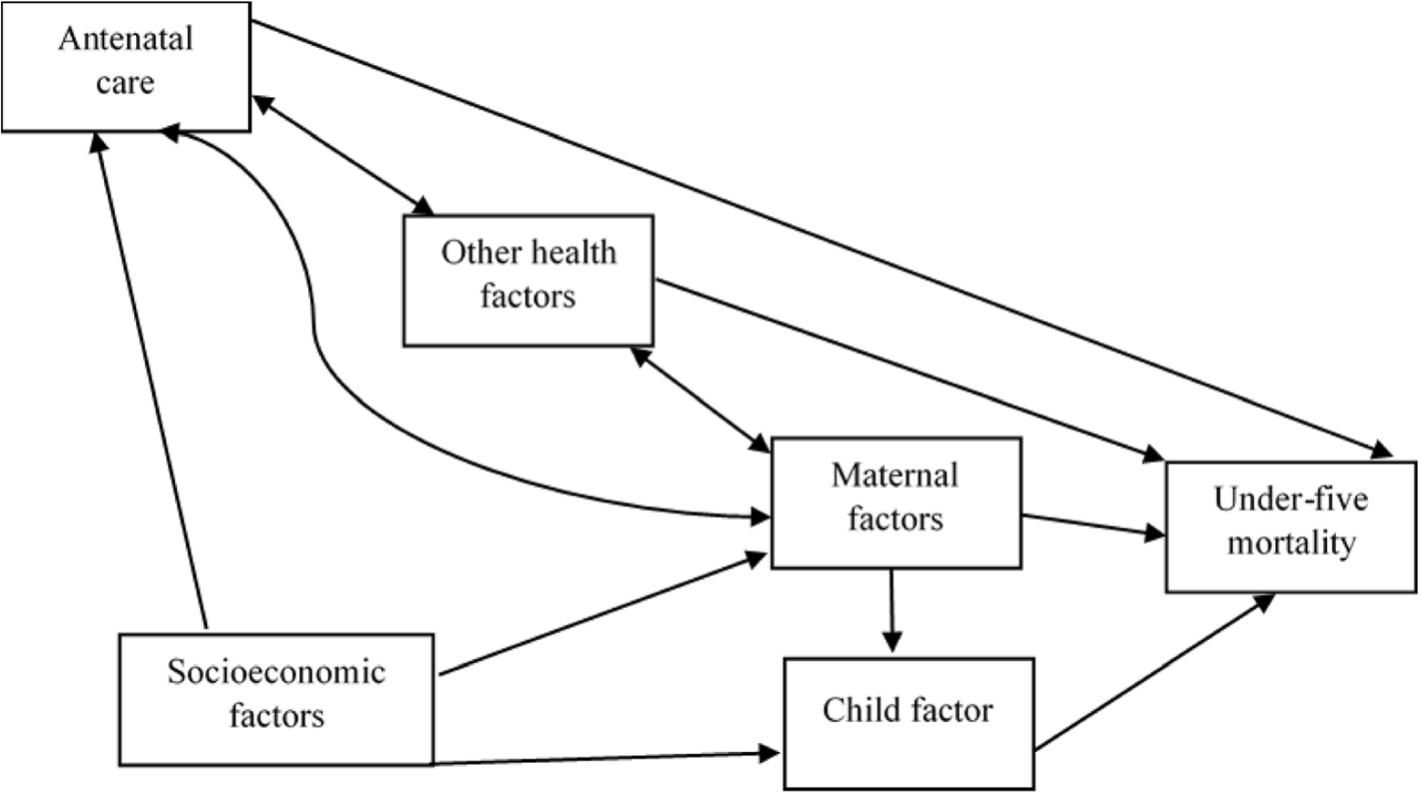
A directed acyclic graph showing the link between antenatal care and under-five mortality.

The treatment (ANC) links with several factors in contributing to under-five mortality. Figure 1 highlights the link between our treatment and the outcome of interest. Socioeconomic factors such as education level, working status, and wealth index are all associated with ANC. Other health factors such as immunization, breastfeeding, and delivery place also interact with ANC in contributing to under-five mortality. These factors also relate to maternal factors such as the age of mothers, religion, age at first birth, and others in linking up with the outcome of interest. Child factors such as birth size, birth order, and preceding birth interval are all associated with socioeconomic factors and maternal factors linked with ANC and under-five mortality.

### Statistical analysis

We adapted a two-stage research design to improve the comparison between the treated and control groups. Our analyses begin by matching individuals with household characteristics that provide an equal probability of utilizing ANCs across groups. To this end, we used the Propensity Score Matching (PSM). The PMS is a statistical technique that seeks to address the primary drawback of causal inferences from observational research designs where no standardized methods have been used to establish control groups [25]. This technique involves forming matched sets of control and treatment of individuals whose propensity scores are similar [26]. If a matched sample has been established, the treatment effect can be assessed by comparing the outcomes directly between treated and control subjects in the matched sample [27].

The propensity scoring matching (PSM) approach was applied to compare under-five mortality among women who utilize ANC and Women who do not [28]. The PSM approach was chosen because ANC utilization is not random and can be affected strongly by observable and non-observable factors.

The criteria for selecting the PSM variables are demographic and socioeconomic covariates from the EDHS that are significantly associated with ANC and child mortality. Women who utilized ANC were matched to women who did not utilize ANC based on a logit regression. We used a Chi-Square test to access the balance for all covariates before and after PSM, with a 5% level of significance or more considered indicative of imbalance.

The Difference-in-Differences (DID) method was used to analyze the effect of ANC on under-five mortality. The DID is a quasi-experimental approach that compares outcome changes over time between a group involved in intervention (treatment group) and a group that is not (control group) [29]. While the DID method typically uses panel data to estimate the causal impact of policies or programs, repeated cross-sectional data from the same areas has also been used in the literature [30–32].

We apply the DID method using the linear probability model:

$${Y}_{it}={\beta }_{0}+{\beta }_{1}{Treatment}_{i}+{\beta }_{2}{Time}_{t}+{\beta }_{3}\left({Treatment}_{i}*{Time}_{t}\right)+{\beta }_{4}{\varvec{X}}_{i}+{\beta }_{5}{\varvec{Z}}_{i}+{\epsilon }_{it}$$

To enable us to estimate the differences in under-five mortality for treatment and control groups. ${Y}_{it}$ in the model refers to the binary indicator of whether a child $i$ born in year $t$ died or not before reaching the age of five (under-five mortality). The variable ${Treatment}_{i}$is a dummy with 1 indicating mother had ANC and 0 otherwise. The variable ${Time}_{t}$ is also a dummy variable coded 0 for 2011 and 1 for 2016. The DID estimate ${\beta }_{3}$ of the effect of ANC, is an interaction between $Treatment$ and $Time$. The vector ${\varvec{X}}_{i}$ is a vector of variables controlled by propensity score matching and ${\varvec{Z}}_{i}$ is a vector of additional covariates to adjust for the remaining imbalance from our matching procedure. To account for the survey design’s complexity, the primary sampling unit, strata, and person weight were incorporated in the regression models to adjust for the standard error. We carried out all statistical analyses using SAS version 9.4.

## Results

### Descriptive of the study data

The crude rates of under-five mortality in 2011 and 2016 are shown in Table 1 for the different number of ANC visits. In 2011 the under-five mortality rate was 5.6% for women who had only one ANC visit, compared to 8.2% who had no ANC visit for their entire pregnancy. In 2016, there was a drop in under-five mortality for women who had only one ANC visit (2.9%), but the rate remains the same in 2016 for women who did not attend any ANC. Considering Treatment 2, under-five mortality was 4.2% in 2011 for women who had just two ANC visits, compared to 8.1% for women who had one or no visit. Under-five mortality decreased to 3.4% for the women with only two ANC visits and 7.9% for women who had just one or no visit.

**Table 1:** Under-five mortality rates for the number of ANC visits treatments and controls

	2011		2016	
	Under-five mortality		Under-five mortality	
	Yes (%)	No (%)	Yes (%)	No (%)
Treatment 1	19 (5.6)	319 (94.4)	10 (2.9)	332 (97.1)
Control 1	641 (8.2)	7162 (91.8)	431 (8.2)	4810 (91.8)
Treatment 2	22 (4.2)	507 (95.8)	19 (3.4)	544 (96.6)
Control 2	660 (8.1)	7481 (91.9)	441 (7.9)	5142 (92.1)
Treatment 3	57 (4.0)	1361 (96.0)	63 (2.7)	2300 (97.3)
Control 3	778 (7.8)	9147 (92.2)	568 (7.1)	7454 (92.6)
Treatment 4	11 (3.5)	300 (96.5)	4 (1.6)	252 (98.4)
Control 4	835 (7.4)	10,508 (92.6)	631 (6.1)	9754 (93.9)

For treatment 3, for women who had four to seven ANC visits, under-five mortality was 4.2% in 2011 and 2.7% in 2016. Comparing that to women who had less than four ANC visits, the under-five mortality rate was 7.8% and 7.1%, respectively, for 2011 and 2016. The under-five mortality rate was lowest for women who had eight or more ANC visits, with the rate been 3.5% in 2011 and 1.6% in 2016. Comparing that for women who had less than eight ANC visits, the under-five mortality rate was 7.4% in 2011 and 6.1% in 2016.

Under-five mortality rates are shown in Table 2 for the timing of the first ANC visit. The under-five mortality rate remains 8.2% for women with no ANC visit in 2011 and 2016, respectively. For women who had their first ANC visit within the first trimester of pregnancy, the under-five mortality rate was 5.5% and 4.4% for 2011 and 2016, respectively. Women who had their first ANC visit within the second trimester had an under-five mortality rate of 5.4% in 2011 and 3.3% in 2016. For women who had their first ANC visit within the third trimester, the under-five mortality rate was 4.4% and 3.3% in 2011 and 2016, respectively.

**Table 2:** Under-five mortality rates for the timing to first ANC visit

	2011			2016	
	Under-five mortality			Under-five mortality	
	Yes	No		Yes	No
No visit	641 (8.2)	7162 (91.8)		431 (8.2)	4810 (91.8)
First trimester	79 (5.5)	1346 (94.5)		103 (4.4)	2256 (95.6)
Second trimester	106 (5.4)	1868 (94.6)		87 (3.3)	2536 (96.7)
Third trimester	20 (4.4)	432 (95.6)		14 (3.3)	404 (96.7)

### Propensity score matching result

The PSM produced a matched sample of 9 124 birth cases. Table 3 contrasts the demographic and socioeconomic characteristics of women with ANC visits and women who did not, before and after propensity score matching. Before PSM, all the baseline characteristics showed no significant difference (p-value < 0.05) in under-five mortality between the treatment and control group.

**Table 3:** Descriptives for individual and socioeconomic characteristic before and after PSM

	% Before PSM				%After PSM	
	ANC visits				ANC visits	
	No	Yes			No	Yes
**Characteristics**	***n* = 13,044**	***n* = 9251**	***p*-value**	***n* = 4562**	***n* = 4562**	***p*-value**
**The child is a twin or single birth**			< 0.001			0.153
Single birth/not a twin	58.4	41.6		50.1	49.9	
1st of multiple births	70.5	29.5		38.2	61.8	
2nd of multiple births	56.8	43.2		43.5	56.5	
3rd of multiple births	0	100.0		0	0	
**Size of the child at birth**			< 0.001			0.390
Very large	54.4	45.6		50.7	49.3	
Larger than average	57.7	42.3		49.7	50.3	
Average	57.1	42.9		50.1	49.9	
Smaller than average	63.9	36.1		46.7	53.3	
Very small	62.8	37.2		50.7	49.3	
**Child’s birth order number**			< 0.001			0.523
1	45.7	54.3		48.7	51.3	
2–4	57.7	42.3		50.1	49.9	
5+	66.7	33.3		50.4	49.6	
**Length of preceding birth interval**			< 0.001			0.520
First birth	45.6	54.4		49.1	50.9	
Less than 12 months	76.4	23.6		50.0	50.0	
12–23 months	71.0	29.0		48.6	51.4	
24 + months	58.8	41.2		50.5	49.5	
**Age of mother**			< 0.001			0.932
15–19	50.7	49.3		49.7	50.3	
20–24	54.2	45.8		49.3	50.7	
25–29	58.2	41.8		49.9	50.1	
30–34	59.8	40.2		50.8	49.2	
35–39	61.5	38.5		50.0	50.0	
40–44	64.4	35.6		51.2	48.8	
45–49	66.8	33.2		47.3	52.7	
**Age at the time of first birth**			< 0.001			0.386
Less than 18 years	62.8	37.2		50.6	49.4	
18–21 years	58.3	41.7		50.1	49.9	
22–29 years	51.8	48.2		48.4	51.6	
30 + years	40.4	59.6		44.0	56.0	
**Highest educational level**			< 0.001			0.961
No education	69.3	30.7		50.0	50.0	
Primary	44.1	55.9		50.1	49.9	
Secondary	14.4	85.6		48.5	51.5	
Higher	5.1	94.9		52.6	47.4	
**Household wealth index**			< 0.001			0.703
Poorest	75.9	24.1		49.9	50.1	
Poorer	65.0	35.0		50.2	49.8	
Middle	60.5	39.5		51.3	48.7	
Richer	55.9	44.1		49.3	50.7	
Richest	21.9	78.1		48.9	51.1	
**Urban-rural status**			< 0.001			1.000
Urban	21.5	78.5		50	50	
Rural	66.5	33.5		50	50	
**Region**			< 0.001			0.791
Tigray	39.4	60.6		51.1	48.9	
Affar	75.9	24.1		49.4	50.6	
Amhara	58.8	41.2		51.2	48.8	
Oromia	68.4	31.6		50.3	49.7	
Somali	75.0	25.0		50.9	49.1	
Benishangul-Gumuz	61.1	38.9		50.2	49.8	
Southern Nations, Nationalities, and Peoples	61.3	38.7		49.9	50.1	
Gambela	57.2	42.8		47.9	52.1	
Harari	45.6	54.4		46.1	53.9	
Addis Ababa	4.6	95.4		54.5	45.5	
Dire Dawa	43.1	56.9		48.6	51.4	
**Religion**			< 0.001			0.669
MUSLIM	65.4	34.6		49.9	50.1	
Catholic	58.0	42.0		46.5	53.5	
Orthodox	44.3	55.7		50.7	49.3	
Protestant	61.0	39.0		49.6	50.4	
Others	81.3	18.7		44.4	55.6	

After the PSM, all characteristics showed a significant (P-value > 0.05) difference in under-five mortality between the treatment and control group, suggesting that the PSM approach significantly reduced the between-group differences in the observed characteristics.

### DID estimation results

Table 3 summarized the DID analysis’s primary outcome for the matched sample when no covariate was added in the model. The estimates in the model for Treatment 1 suggest that for a woman to have one ANC visit reduces the likelihood of under-five mortality by 6.0% (CI = 1.9–13.9%, P-value = 0.135). However, this estimate was not significant, as the p-value fall above 0.05. Therefore, we can say there is no significant difference in under-five mortality for women who had just one ANC visit and women who did not attend ANC at all. Similarly, under-five mortality was 3% less for women who had two ANC visits but was not significantly (CI = 3.2–10.6%, P-value = 0.295) different from women who had one or no ANC visits. Also, women who had between 4 and 7 ANC visits had a 1.5% (CI= -0.4-6.4%, P-value = 0.548) lower likelihood of under-mortality than women who had less than four visits. Still, again the effect was not statistically significant. Finally, from Table 4, women having eight or more ANC visits reduces the likelihood of under-five mortality significantly by 39.0% (CI = 23.3–54.7%, *P*-value < 0.001).

**Table 4:** The DID for the number of ANC visits with no covariates in the models

Treatment effect	Estimate	95% CI		t Value	*P*-value
		Lower	Upper		
ANC 1 visit	-0.060	-0.139	0.019	-1.500	0.135
ANC 2 visits	-0.037	-0.1061	0.032	-1.050	0.295
ANC 4–7 visits	-0.015	-0.064	0.034	-0.600	0.548
ANC 8 + visits	-0.390	-0.547	-0.233	-4.870	< 0.001

We adjusted for the effect of immunization, breastfeeding, and delivery, and the results for the models are shown in Table 5. After adjusting for the covariates, the impact of the number of ANC visits on under-five mortality remains insignificant for those with 1, 2, and 4–7 ANC visits. However, having eight or more visits reduces the likelihood of under-five mortality by 45.2% (CI = 19.2–71.3%, *P*-value < 0.001) after adjusting for immunization, breastfeeding, and place of delivery.

**Table 5:** The DID for the number of ANC visits adjusting for immunization, breastfeeding, and place of delivery

Treatment effect	Estimate	95% CI		t Value	*P*-value
		Lower	Upper		
ANC 1 visit	-0.012	-0.114	0.090	-0.23	0.821
ANC 2 visits	-0.004	-0.098	0.089	-0.09	0.930
ANC 4–7 visits	-0.040	-0.110	0.031	-1.11	0.268
ANC 8 + visits	-0.452	-0.713	-0.192	-3.40	< 0.001

Using the adjusted model, we estimated the effect of the first ANC visit time on under-five mortality, and the results are shown in Table 6. The first visit within the third trimester shows no statistically significant (*P*-value = 0.894) effect on under-five mortality compared to having no ANC visit. Having the first visit within the second trimester decreases the likelihood of under-five mortality significantly by 6.7% (CI = 2.4–11.0%, *P*-value = 0.002) compared to those in the third trimester or had no visit. Women who had their first ANC within the first trimester had 10% (CI = 5.7–15.6%, *P*-value < 0.001) less likelihood of under-five mortality than those who had it later than the first trimester.

**Table 6:** The DID for the timing of the first ANC visit

Treatment effect	Estimate	95% CI		t Value	*P*-value
		Lower	Upper		
Third trimester	0.006	-0.079	0.090	0.130	0.894
Second trimester	-0.067	-0.110	-0.024	-3.040	0.002
First trimester	-0.107	-0.156	-0.057	-4.240	< 0.001

## Discussion

Using data from extensive nationally representative demographic and health surveys, we investigated the impact of ANC visits and timing of first ANC visit on child health outcomes, specifically under-five mortality. The study rate of under-five mortality dropped from 7.3% in 2011 to 6.0% in 2016. Based on a DID model, we present evidence that 45.2% of the decrease in under-five mortality resulted from having eight or more ANC visits, and 10.7% of the reduction in under-five mortality resulted from having the first ANC visit within the first trimester.

To the best of our knowledge, this study is one of the first studies investigating the causal impact of the number of ANC visits and the timing of the first ANC visit on under-five mortality. However, several cross-sectional studies have associated increased ANC visits with a significant decrease in child mortality [33–35]. In particular, Jana Kuhnt and Sebastian Vollmer [36] used national representative health and welfare data from 193 Demographic and Health Surveys conducted between 1990 and 2013 to investigate antenatal care implication on the health outcome of children. They found using linear probability regression that at least one ANC visit was associated with a 1.04% decrease in risk of neonatal mortality and a 1.07% decrease in risk of infant mortality. Another cross-sectional study in Bangladesh by Tanvir et al. [37] used multivariate logistic regression to analyze three demographic health surveys and found the odds of under-five mortality to be lower for women with ANC visits. Malachi et al. [38] investigated the effectiveness of antenatal care services in reducing neonatal mortality in Kenya. Their findings using a binary logistic regression show the lowest neonatal mortality rates to belong to mothers who attended ANC visits.

The Ethiopian government has been putting up measures to improve maternal and child health outcomes, such as providing free health care services for the poor, creating ANC centers across the country, and making primary health care accessible for all [16, 39]. Past studies in Ethiopia have identified factors such as urban residence, higher educational attainment, wealth, perceived good quality of maternal health services, and mass media exposure to be significantly associated with increased ANC visits [40–42]. The finding of this study will assist policymakers in seeing the real impact of ANC visits on under-five mortality. That will guide in intensifying the advocacy of ANC.

### Strengths and limitations

One major strength of this study is the use of nationally representative data. Although this was not a randomized control trial, a repeated cross-sectional survey from the same sample frame can be used to obtain causal inference [43]. The double approach we employed ensures that bias is reduced to the minimum by matching the treatment to the control to be similar before applying the quasi-experimental study design for causal inference.

The DID approach assumes that individuals’ unmeasured critical characteristics could affect the outcome based on differences between the treatment and control group [44]. As mentioned above, we reduced possible bias to the minimum by using a double approach, but we cannot completely rule out unmeasured characteristics. However, our selection of factors was informed through a careful study of literature. Another limitation is the quality of data. As peculiar to most national surveys, there will be the presence of missingness in the data. This problem was addressed using multiple imputations. This technique is efficient where data are missing at random, and we ensured that the imputed values were predictive of the missing values. There was no significant difference in our results before and after the imputation.

## Conclusions

This paper examined the effects of antenatal care on under-five mortality using propensity score matching and difference-in-difference logistic regression analysis. We found evidence suggesting that the number of ANC visits and the timing of the first ANC visit have an impact on under-five mortality, and we recommend eight or more visits with the first visit being within the first trimester of gestation; this is in line with the 2016 WHO guideline. Intervention programs that encourage ANC visits should also be considered if meaningful progress is to be achieved to reduce under-five mortality and realize the Sustainable Development Goal by 2030. We further recommend a study that will examine the causal impact of ANC on under-five mortality extensively.

## Data Availability

The dataset supporting the conclusions of this article is available in the IDHS repository. https://www.idhsdata.org/idhs-action/menu.

## Abbreviations

ANC: Antenatal care
C I: Confidence Interval
DID: Difference-in-Differences
EDHS: Ethiopian Demographic and Health Survey
PSM: Propensity score matching
P-value: Probability Value
